# Polysacharide of *Agaricus* *blazei* gel mitigates bone necrosis in model of the jaws related to bisphosphonate via Wnt signaling

**DOI:** 10.1038/s41598-024-58445-5

**Published:** 2024-04-08

**Authors:** Vanessa Costa de Sousa, Fátima Regina Nunes Sousa, Raquel Felipe Vasconcelos, Gisele Angelino Barreto, Conceição S. Martins, Nilson Romero Dias, Sislana Costa, Maria Jennifer Chaves Bernardino, George de Almeida Silva, Nadine Linhares, Delane Gondim, Mirna Marques, Helliada Chaves, Karuza Alves, Renata Leitão, Gerly A. C. Brito, Maria Elenir Nobre Pinho Ribeiro, Paula Goes

**Affiliations:** 1 Post Graduation of Morphological Science, Department of Morphology, Medical School, Federal University of Ceará, Fortaleza, CE Brazil; 2 Medical School, Federal University of Piauí, Picos, CE Brazil; 3 Federal Institute of Ceará, Fortaleza, CE Brazil; 4 Department of Morphology, Medical School, Federal University of Ceará, Fortaleza, CE Brazil; 5 Department of Chemistry, Federal University of Ceará, Fortaleza, CE Brazil; 6 Post Graduation of Dentistry, School of Dentistry, Federal University of Ceará, Fortaleza, CE Brazil; 7 Faculty of Medical School, Federal University of Ceará, Sobral, CE Brazil; 8 School of Dentistry, Federal University of Ceará, Sobral, CE Brazil; 9 Department of Pathology and Legal Medicine, Medical School, Federal University of Ceará, Fortaleza, CE Brazil; 10 Lab of Medical Immunology, Department of Pathology and Legal Medicine, Faculty of Medicine, Federal University of Ceará, Rua Coronel Nunes de Melo, 1315, Rodolfo Teófilo, Fortaleza, CE 60430-270 Brazil

**Keywords:** BRONJ, Beta-glucan, Osteoblast, Wnt signaling, Bone remodeling, Bone quality, Immunology, Medical research, Pathogenesis

## Abstract

To investigate de effect of PAb gel on the bone tissue of rats submitted to Bisphosphonate-related osteonecrosis of the jaws (BRONJ). Initially, 54 animals were submitted to BRONJ model by Zoledronic Acid (ZA) (0.1 mg/kg 3x/wk for 9 wk, ip), followed by the 1st upper left molar extraction at the 8th wk. After tooth removal, the animals were divided into 3 groups, ZA that received placebo gel or PAb gel that received 1% PAb gel, inside the dental alveolus. The control Group (CONTROL) received 0.1 mg/kg of 0.9% saline and then placebo gel. Three weeks after tooth extraction, the animals were euthanized, and maxillae were colleted for macroscopic, radiographic, histological and Raman spectomery assays. Additionally, GSK3b, beta-catenin, and Runx2 mRNA expressions were determined. Blood samples were collected for the analysis of Bone-specific alkaline phosphatase (BALP) levels. PAb gel improved mucosal healing, increased the number of viable osteocytes, while it reduced the number of empty lacunae, as well as the amount of bone sequestration. Furthermore, PAb gel positively influenced the number and functionality of osteoblasts by stimulating Wnt signaling, thereby inducing bone remodeling. Additionally, PAb gel contributed to improved bone quality, as evidenced by an increase in bone mineral content, a decrease in bone solubility, and an enhancement in the quality of collagen, particularly type I collagen. PAb gel mitigated bone necrosis by stimulating of bone remodeling through Wnt signaling and concurrently improved bone quality. PAb gel emerges as a promising pharmacological tool for aiding in BRONJ therapy or potentially preventing the development of BRONJ.

## Introduction

Medication-related osteonecrosis of the jaw (MRONJ) is a rare yet severe condition that can affect the upper or lower jaw^1^. The American Association of Oral and Maxillofacial Surgeons (AAOMS) introduced this term to encompass the increasing number of osteonecrosis cases involving the maxilla and mandible that are associated with other antiresorptive and antiangiogenic therapies^2^. Among the different types of MRONJ, bisphosphonate-related osteonecrosis of the jaw (BRONJ) has demonstrated a higher prevalence rate^3^, ranging from 1.6 to 14.8% when intravenous bisphosphonates were used followed by tooth extraction^4^.

The pathophysiology of BRONJ is complex and multifactorial. Although clinicians and researchers have engaged in extensive discussions about the etiological factors of BRONJ, several key factors contribute to the development of this condition, including bone remodeling inhibition, inflammation or infection, angiogenesis inhibition, innate or acquired immune dysfunction, and genetic predisposition^2^. When examining bone remodeling in more detail, it is well-established that osteoclast inhibition plays a central role in this process. However, the involvement of osteoblasts and osteocytes in BRONJ is not as thoroughly explored^5^. Our research group has recently demonstrated a reduction in osteoblast numbers in a rat BRONJ model, linked to the inhibition of Wnt signaling^6^. As a result, therapeutic strategies that target osteoblastogenesis could offer a promising approach to managing BRONJ.

The primary objective of BRONJ therapy is to prevent the onset of the disease by optimizing dental health and avoiding dentoalveolar surgical procedures. Additionally, international professional societies have recommended the use of drug holidays, although strong evidence supporting their effectiveness is lacking. Once the disease is established, the use of chlorhexidine and systemic antibiotics has been proposed, but it does not always lead to complete resolution and has limited long-term effectiveness. Furthermore, the removal of necrotic bone through surgical procedures is typically recommended, particularly in more advanced stages of the disease. Adjunct therapies, such as hyperbaric oxygen or ozone therapy, as well as the use of vitamin E and pentoxifylline, have been studied. However, highly effective treatment protocols are yet to be determined^2^.

The urge of effective therapeutical approaches for BRONJ has stimulated the development of new biomaterials. *Agaricus blazei* (Ab) is a mushroom of the *Basidomycota* family that grows freely in Brazil. Ab has mainly been used by the local population as a food ingredient, but also as a medicine against a wide range of diseases, in particular infection and cancer^7^. The fruiting body of Ab is rich in β-glucans, characterized by chains of D-glucose linked by β-type glycosidic bonds, being β- (1–3) linked backbone with (1–6) linked side branches^8^. Biologically, this polysaccharide has shown immunomodulating effects^9,10^. On bone tissue, beta-glucan has shown antiresorptive effects in experimental periodontitis model^11,12^ and positive effects on bone regeneration and metabolism^10,13^. However, no study has ever investigated the effect of the polysaccharide beta-glucan derived from *Agaricus blazei (PAb)*, neither topically nor systemically, on BRONJ.

In the context of addressing the need for effective therapeutic approaches for BRONJ, there has been a growing interest in the development of new biomaterials. Agaricus blazei (Ab) is a mushroom belonging to the Basidiomycota family and is commonly found in Brazil. While Ab has traditionally been used as a food ingredient by the local population, it has also been employed as a remedy for a wide range of diseases, particularly for infections and cancer^7^. The fruiting body of Ab is rich in β-glucans, which are characterized by chains of D-glucose linked by β-type glycosidic bonds, with a β-(1–3) linked backbone and (1–6) linked side branches^8^. Biologically, this polysaccharide has demonstrated immunomodulatory effects^9,10^. On bone tissue, beta-glucan has shown antiresorptive effects in experimental periodontitis models^11,12^ and has had positive effects on bone regeneration and metabolism^10,13^. However, to date, no study has investigated the effects of the polysaccharide beta-glucan derived from Agaricus blazei (PAb), whether administered topically or systemically, on BRONJ.

Hence, given the promising and beneficial effects that PAb has demonstrated on bone metabolism, and considering that BRONJ is primarily linked to the inhibition of bone remodeling, we have hypothesized that PAb gel can stimulate bone formation and alleviate BRONJ in rats. This emphasizes the application of β-glucans as a biocompatible strategy and a potential candidate for the management of bone-related diseases.

## Materials and methods

### Study design and ethical aspects

This was a pre-clinical randomized and blinded study. The experiments were only initiated after approval by the Institutional Ethics Committee for Animal Research Federal University of Ceará (UFC) (number 4411060619).

All methods were performed in accordance with the relevant guidelines and regulations described in ARRIVE guidelines.

### Animals and experimental groups

For this study, 54 female Wistar rats (12 weeks old,  ± 200 g) (*Rattus novergicus)* were used. The sample size of 6 animals per group was determined in order to provide a power calculation of 80%, and significant level of p < 0.05, considering bone necrosis, defined by the percentage of empty lacunae of osteocytes (> 50% in 05 fields/slide) and presence of bone sequestration as the primary outcome variable^6,14^.

Throughout the whole experiment the animals were kept in cages (n = 3 animals/cage) at temperature-controlled rooms, with free food and water. After two weeks of acclimation to the laboratory environment, the animals were divided in a blind and randomized manner. Randomization was performed by computer software, considering the weight of the animals. Three experimental groups were established as follows:

- Control group: where the animals received 0.1 mg/kg 0.9% saline solution 3x/wk intraperitoneally (ip) for 09 weeks and then placebo gel in the dental alveolus;
- Zoledronic acid (ZA) group: the animals were submitted to bisphosphonate-related osteonecrosis of the jaws (BRONJ) model, and then placebo gel in the dental alveolus;
- Polyssacharide of *Agaricus blazei* (PAb) gel group: the animals were submitted to BRONJ model, and then received 1.0% PAb gel in the dental alveolus;

The gels used in this study (placebo and 1.0% PAb) were inserted in the dental socket, after tooth extraction using a hypodermic needle (25 × 0.8 mm) previously prepared, pre-crooked and without bevel, in a single administration^15^.

The study was divided into 3 sets of experiments: in the 1st set, the collected maxillae were used for macro and microscopic analyses. In the 2nd set the maxillae were used for radiographic and micro-Raman spectroscopy and the 3st for PCR in time real. All analyses were performed by an experienced examiner unaware of the groups.

### BRONJ-like model

**Figure 1:**
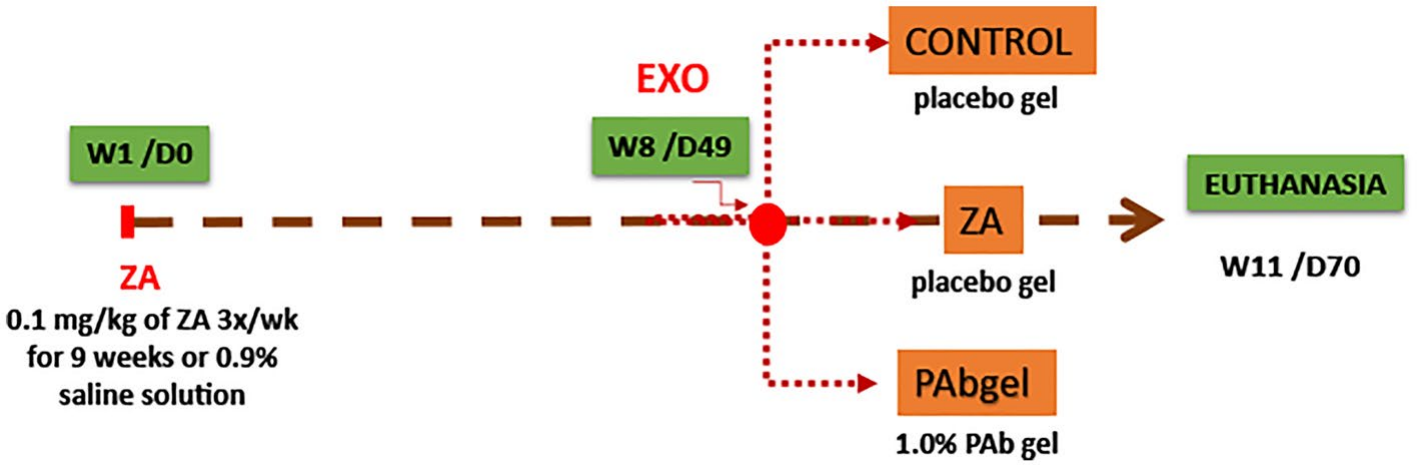
Experimental study design. Animals were divided into 3 groups, control, ZA and PAb gel groups. In the Control group, the animals were not submitted to BRONJ. They received a saline solution, and tooth extraction was carried out (D49/S8) and a placebo gel was placed on the dental socket. In the ZA group, the animals received AZ (0.1 mg/kg-i.p.), and the tooth extraction was carried out (D49/S8). In PAb group, the animals were submitted to BRONJ model, and immediately after tooth extraction, the animals received 1.0% of PAb gel the dental socket in. All gels were administered in a single application. The animals were followed until the day of euthanasia (D77/S11). *ZA*   zoledronic acid, *PAb*  polyssacharide of *Agaricus* *blazei*, *w* week, *d* day, *EXO*   exodontia.

It was used a BRONJ-like rat model previous reported by de Sousa Ferreira et al.^6^. The animals received 0.1 mg/kg of Zoledronic Acid (Cristália, Itapira, SP, Brazil) intraperitoneally (ip) 3x/wk for 9 weeks^6,16^. On the 8th week (49th experimental day—D49/W8), all animals, previously anesthesized with ketamine and xylazine ip., were submitted to the extraction of 1st upper left molar [Refs.^6,16^ with modifications]. Three weeks after tooth extraction the animals were euthanized by overdose of anesthetics (Fig. 1).

### Preparation of *Agaricus blazei*-glucan polysaccharides (PAb) gel

Initially, 2.0 ml of a diluted graphene oxide (GOx) solution at a concentration of 0.75 mg/mL was prepared, then placed in an ultrasound bath for 5 min, undergoing a stirring fast and strong process. Following it was performed the incorporation of the active ingredient: it was added 20.0 mg of *Agaricus blazei* -glucan polysaccharides (PAb) register Sisgen number AC29F45 to GOx solution to the GOx solution. The gels were stirred overnight at room temperature, then 50.0 mg of Hydroxypropylmethylcellulose (HPMC) were added to the previously prepared suspension, obtaining the hydrogel (2.5% w/v of HPMC) according to the gel to be produced. Finally, a process of intense agitation was successfully carried out, followed by cooling at 5 °C for 3 days to obtain a homogeneous phase of polymer, solvent and drug. The gels were prepared after investigating the physical properties, specificity, viscosity, kinetics and stability of the drug, in the following concentrations: 1.0% *Agaricus blazei* -glucan polysaccharides Gel (1.0% w/v PAb)^17^.

### Macroscopic analysis

After euthanasia, detailed visual inspection of the maxillae was performed. Macroscopically, it was evaluated the presence or absence of bone exposure, as well as the oral mucosa continuity solution in the extraction region^6^. Data was presented in frequency.

### Radiographic density analysis

For radiographic density analysis maxillae were radiographed by Digora® (Soredex, Finland), and then evaluated with Image J 1.31 software (ImageJ 1.32j, National Institute of Health, USA). A region of interest (ROI) consisting of 128 pixels was selected in the extraction site (considering as the upper limit the cervical of the second molar extending in the apical direction). The grayscale differences of both areas were considered radiographic density values. The analysis of the radiographic density of the ROI was performed using the histogram tool from Image J^®^ software, using 256 shades of gray scale, where zero indicates black and 255 white. Data were expressed in arbitrary shades of gray^18^.

### Histopathological analysis

After macroscopic and radiographic analyses, maxillae were demineralized in 10% EDTA buffered solution. After 30 days of decalcification, the specimens were embedded in paraffin. Serial sections of 4 µm thickness, representing the area around the alveolar socket, obtained in a mesiodistal direction, were stained with hematoxylin and eosin (HE) for histopathological and histomorphometric analysis. The histopathological analysis was performed, at 100 × magnification and the presence of bone sequestration was evaluated using scores where 0 indicates absence and 1 indicates^6,19^. These parameters were presented as median and extreme values.

Cells counts were also performed. Ten fields of HE stained slide, adjacent to the extraction site, were captured at 400 × magnification. In the same field, the number of osteocytes and empty lacuna/bone surface were counted as well as the number of osteoblast/bone perimeter (N.Ob./B.Pm.) using Image J® software (NIH, Bethesda, MD, USA) using the cell contain command^20^. The results were expressed as mean ± S.E.M.

Another section of the previously obtained paraffin block was collected for picrosirius red staining. The slides were analysed under a normal and polarized light filter. The quantitative estimation of collagen birefringence, as yellow–red for type I collagen and green for type III collagen^21^, was determined from digital images of 6 fields of each section (from 6 specimens per group), at 200× magnification, using ImageJ^®^ software, according to de Sousa Ferreira et al.^6^. Data was presented as the mean percentage ± S.E.M. of collagen content per group. The counts were performed using Image J 1.51 j8 software (NIH, Bethesda, MD, USA) and the data expressed as mean ± S.E.M.^6,19^. Data is presented as mean percentage ± S.E.M.^6,14^.

### Raman microspectroscopy

Micro Raman spectrometry was used to evaluate bone composition and remodeling. For that, samples were placed in a Micro-Raman spectrophotometer (XploRATM, Horiba JobinYvon, Paris, France) coupled to a Confocal microscope (model XploRATM, manufactured by Horiba JobinYvon). Three spectra of each sample were collected. For the standardization acquisitions were carried out in two distinct points, inside and outside the dental alveolus^6,14^. The data were obtained by a LabSpec 6 software data acquisition command system (Horiba, JobinYvon, Paris, France) and analyzed by the Origin 9 program (Originlab© Corporation, One Roundhouse Plaza, Northampton, MA 01060, USA). For a better understanding the change on bone tissue the ratio of the bands was calculated, as follows:

1. Mineral-to-matrix ratio (MTMR) (~ 960 cm^–1^/1454 cm^–1^): indicates the amount of bone mineralization;
2. Carbonated-to-phosphate ratio (CTPR) (~ 1070 cm^–1^/ ~ 960 cm^–1^): indicates “B-type” carbonate substitution and it is related to bone solubility.
3. Mineral maturity ratio (~ 1030 cm^−1^/ ~ 1020 cm^−1^): reflects proportion of apatitic domain compared with non-apatitic surface domain and it is related to bone aging^22^.
4. Collagen crosslinks ratio (1660 cm^−1^/1690 cm^−1^): measures secondary structures of collagen indicating deterioration of collagen structural integrity^23^.
5. HA carbonate/amide I (~ 1070 cm^−1^/ ~ 1667 cm^−1^): used for remodeling evaluation^14,24^.

### RNA isolation and quantitative PCR

In another set of experiments, after euthanasia, the maxillae were collected, the gingival tissue removed, and the bone tissue was macerated in liquid nitrogen using Trizol (Thermo Fischer-Waltham, Massachusetts, USA). The extracted mRNA was quantified using Nanodrop (Thermo Fischer-Waltham, Massachusetts, USA) and then transcribed using Superscript II (Invitrogen). Subsequently, the RT-PCR assay was carried out using SYBR_green as a reference (ABI 7500 Fast; Applied Biosystems). The PCR condition was 50 °C for 2 min and 90 °C for 10 min, then 40 cycles at 95 °C for 15 s and 60 °C for 1 min, where the RT-PCR system at 7900HT from Applied Biosystems. To calculate the results obtained, the threshold cycle method^25^ was used, where they were presented as relative fold increase related to beta-actin. Primer sequences were as following: ß-actina s: TGAGCTGACCAGTTCCCTCT, ß-catenin as: AAGCTCGCTCCTGTGAGTTC; Runx2 s: CCTTCCCTCCGAGACCCTAA, Runx2 as: ATGGCTGCTCCCTTCTGAAC; GSK3b s: AGAAGAGCCATCATGTCGGG; GSK3b as: CCAAAAGCTGAAGGCTGCTG.

### Determination of bone formation

Before euthanasia, 3 ml of blood samples were obtained from the abdominal aorta from all animals, previously anesthetized. The samples were distributed in tubes with clot accelerator for biochemical parameters and sent to Laboratory of Clinical and Toxicological Analysis (LACT) of the of the Federal University of Ceará (UFC). Serum levels of Bone Alkaline Phosphatase (BALP) for analysis of bone formation. Data was presented was expressed as mean ± S.E.M.

### Statistical analysis

The normality of the data was verified through the Shapiro–Wilk test. Parametric data were presented as mean ± standart error of the mean (S.E.M) after ANOVA followed by Tukey test. Non-parametric data were presented as Median (extreme values) after Kruskal Wallis and Dunn’s Tests. In all situations, the significance level of p < 0.05 was adopted. The software used for all analyzes was IBM^®^ SPSS^®^ statistics 20 and charts constructed using GraphPad Prism^®^ version 6.0.

### Ethics approval

Approval was obtained from the ethics committee of Animal Research Federal University of Ceará (UFC) (number 4411060619).

## Results

### PAb gel protected bone tissue of rats submitted to BRONJ

**Figure 2:**
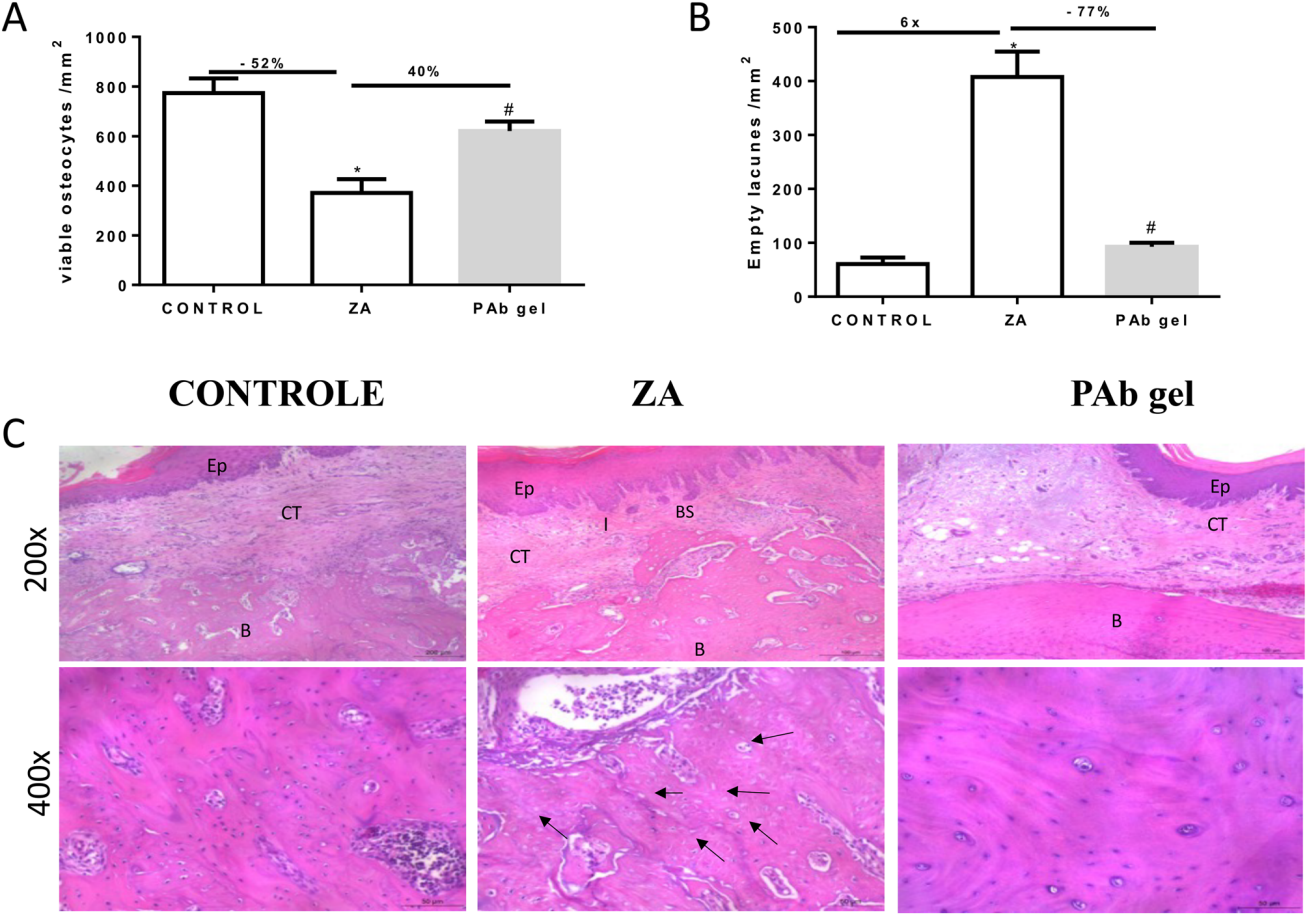
PAb gel protected bone tissue. (**A**) Number of viable osteocytes/mm^2^ (N.Ocy./B.Ar.); (**B**) Number of empty lacunae/mm^2^ (N.EL/B.Ar.); (**C**) Histological aspect of hemimaxillae. Bars represent the mean ± SEM of 6 animals per group. (*) indicates a significant difference when compared to the Control group. (#) indicates a significant difference when compared to the ZA group. Hematoxylin and Eosin (H&E). (200 × and 400 × magnification). Black arrows indicate empty gaps. *Ep* epithelium, *CT* connective tissue, *B* bone, *BS* bone sequestration. ANOVA and Tukey.

The model of BRONJ was effective, once the animals treated with ZA and submitted to tooth extraction presented a significant reduction of viable osteocytes (-52%) (Fig. 2A,C) with a 6 time increase in empty lacuna (Fig. 2B,C) compared to control. Bone sequestration was also seen in ZA group (Table 1). PAb gel, in the other hand, reversed these findings (p < 0.05), considered hallmarks of osteonecrosis.

**Table 1:** Histopathological analysis of PAb on the presence of bone sequestration/bone necrosis.

Bone sequestration/bone necrosis	CONTROL	ZA	PAb gel
(0) missing	05 (83%)	01(17%)	04 (67%)
(1) present	01 (17%)	05 (83%)	02 (33%)^#^
Median (extreme values)	0 (0–1)	2 (0–1)*	0 (0–1)^#^

Values are resented in Median (extreme values) of 6 animals per group. Kruskal–Wallis test followed by Dunn.

*ZA* zoledronic acid, *PAb* polyssacharide of *Agaricus blazei.*

*Indicates difference when compared to the control.

^#^Indicates difference when compared to ZA group (P < 0.05). Data expressed as absolute frequency (percentage frequency).

### PAb gel mitigated BRONJ-like lesion in rats

**Figure 3:**
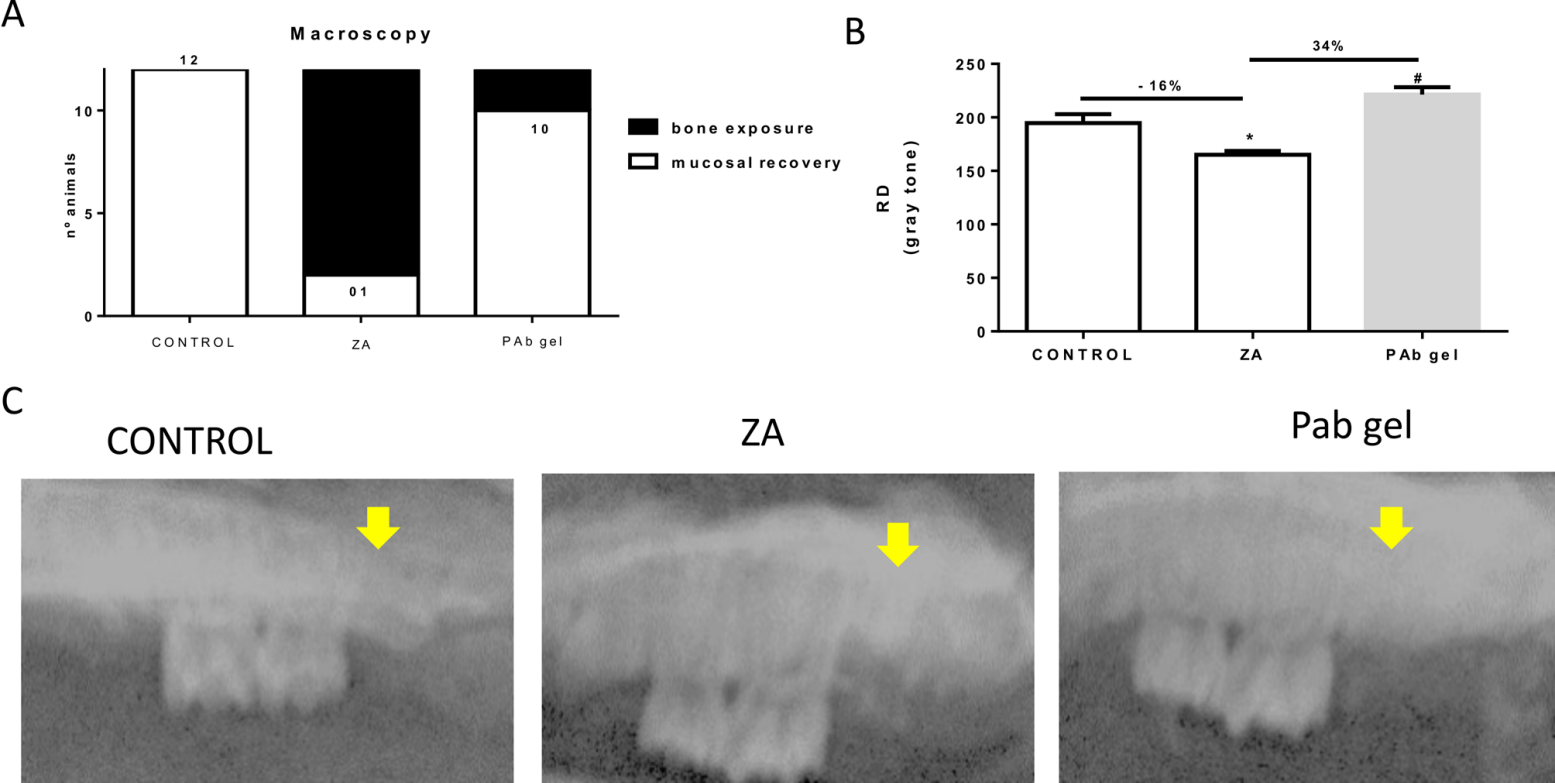
Pab gel mitigated BRONJ-like lesions. (**A**) Distribution of animals considering the macroscopic aspect of bone exposure and mucosal healing in hemimaxillae. Bars represent the mean ± SEM of 12 animals per group. (**B**) Radiographic density (RD) of the hemimaxillae; (**C**) Macroscopic and radiographic aspects of hemimaxilla. (*) indicates a significant difference when compared to the Control group. (#) indicates a significant difference when compared to the ZA group. Yellow arrows indicate radiographic density in the extraction site. ANOVA and Tukey.

BRONJ-like lesions were evaluated by macroscopic and radiographic analyses. Macroscopically, BRONJ was marked by exposure of necrotic bone without mucosal healing (Fig. 3A). Radiographically, it was seen a reduction in the radiographic density in the site of extraction (p < 0.05) compared to control (Fig. 3B and C). Meanwhile, PAb gel promoted mucosal healing and reduced necrotic lesion in maxillary bone (Fig. 3A). The treatment also increased, by 34%, the radiographic density in the area of dental alveolus when compared to ZA group (p < 0.05) (Fig. 3B and C).

### PAb gels stimulated osteoblasts through Wnt signaling

**Figure 4:**
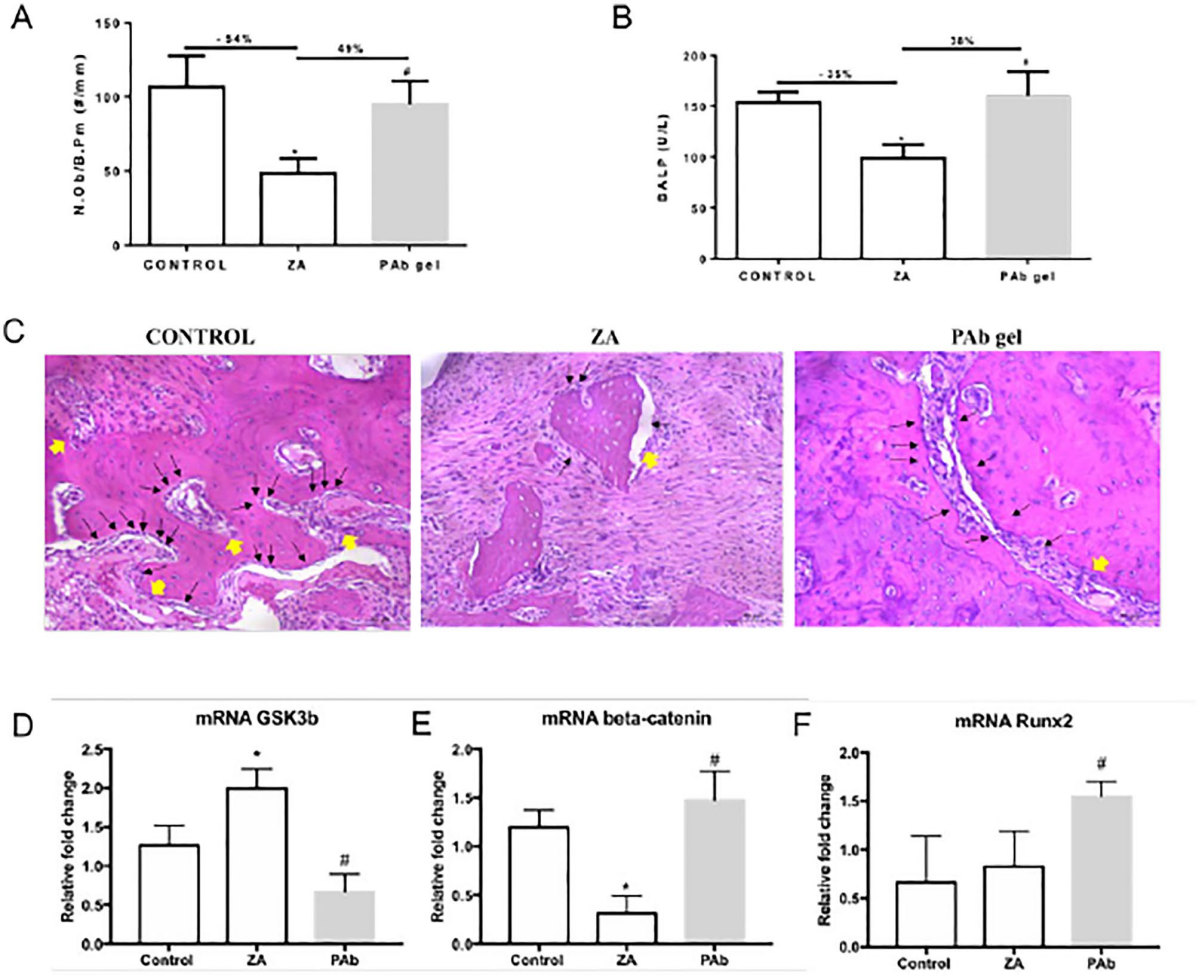
PAb gel stimulate osteoblastogenesis through Wnt signaling. (**A**) Number of osteoblast/bone perimeter (N.Ob./B.Pm.); (**B**) Serum levels of Bone-specific Alkaline Phosphatase (BALP); (**C**) Histological aspect of hemimaxillae; mRNA expression of (**D**) GSK3b; (**E**) Beta-catenin; (**F**) Runx2. Bars represent the mean ± SEM of 6 animals per group. (*) indicates a significant difference when compared to the Control group. (#) indicates a significant difference when compared to the ZA group. Hematoxylin and Eosin (H&E). (200 × magnification). ANOVA and Tukey.

Considering that delayed bone remodeling contributes do osteonecrosis and that osteoblasts plays a role on this process, we have decided to investigated if PAb gel would positively improve this cell activity somehow. Initiialy, we showed that ZA significantly reduced osteoblast count (Fig. 4A and C) and function (Fig. 4B) compared to control. However, when PAb gel was used it was seen a significant increase in both osteoblast number (Fig. 3A and C) and in BALP serum levels, a marker of osteoblast activity (Fig. 4B).

In order to understand the mechanism underlying the benefitial effect of Pab on osteoblast we performed the analysis on the expression of GSK3b and beta-catenin, main players of Wnt signaling, an important pathway related to osteoblastogenesis (Fig. 4D and E). As expected, ZA increased expression on GSK3b mRNA and decreased beta-catenin mRNA expression. Despite the negative impact on Wnt signaling, no significant reduction in the expression of Runx2, a marker of osteoblasts (Fig. 4F). On the contrary, PAb gel to stimulate Wnt signaling, due to the decrease in GSK3b mRNA expression coupled with a significant increased on beta-catenin mRNA expression. A marked increase in Runx2 mRNA expression was seen in the animals with BRONJ treated with PAb gel confirming the findings from histomorphometric analysis.

### PAb gels improve bone quality in BRONJ model

**Figure 5:**
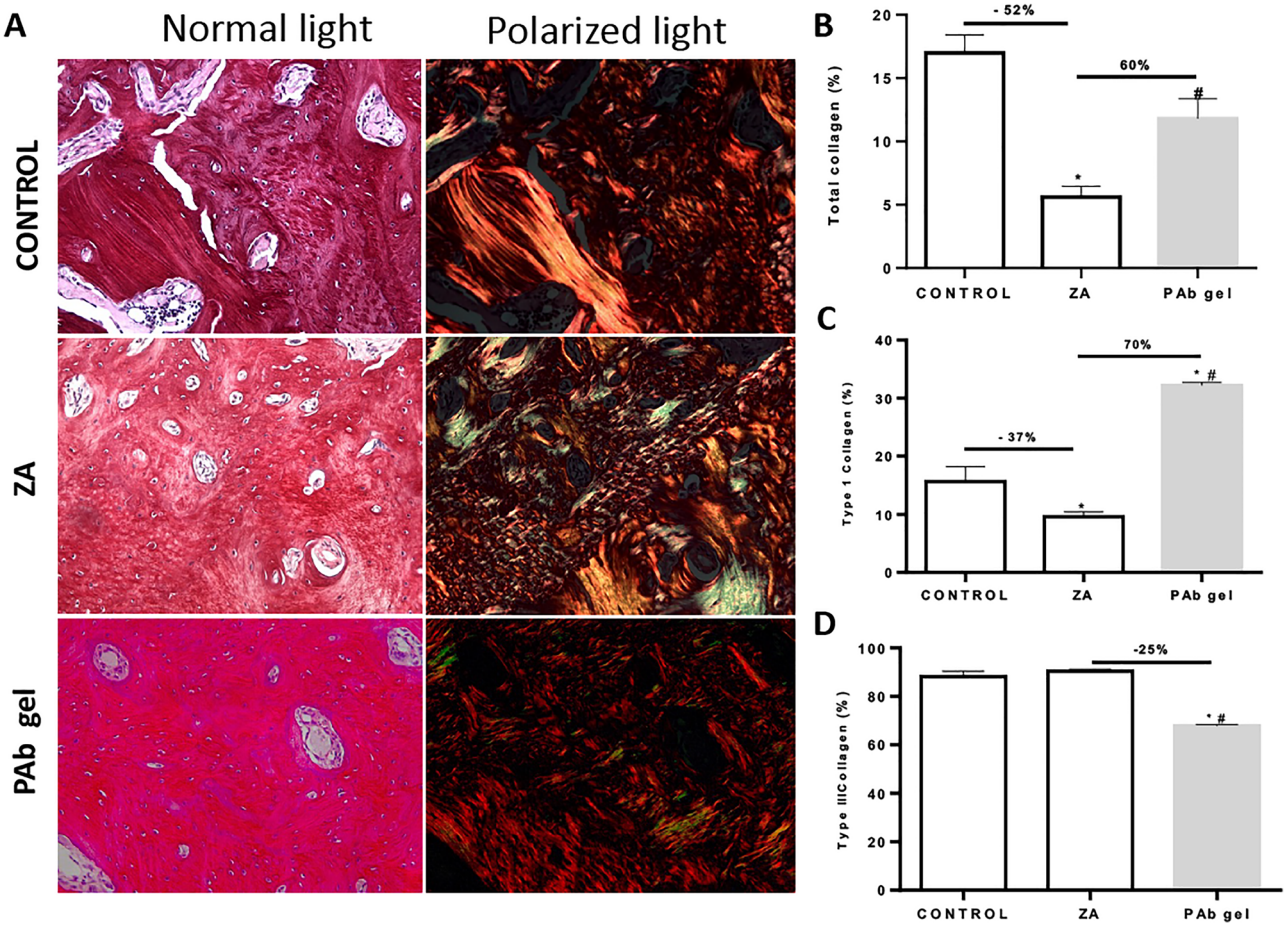
PAb gel improves bone quality. (**A**) Histological aspect of Picrosirius red stained hemimaxillae under normal light and polarized light; (**B**) Percentage of total collagen content on bone tissue; (**C**) Percentage of type I collagen on bone tissue; (**D**) Percentage of type III collagen on bone tissue. Bars represent mean ± SEM of 6 animals per group. Yellow–red indicates type I collagen and green indicates type III collagen. Yellow–red indicates type I collagen and green indicates type III collagen. (*) indicates difference when compared to the Control. (#) indicates difference when compared to ZA group. 200 × magnification. ANOVA and Tukey.

Bone quality was assessed by collagen analysis and Raman spectrometry. The animals receiving ZA and subjected to tooth extraction presented a significant decrease (by 52%) in the amount of total collagen when compared to Control (Fig. 5A and B). This reduction was marked by the decrease on type I collagen on ZA group (37%) (p > 0.05) (Fig. 5C) (p < 0.05). No difference was observed regarding type III (Fig. 5D) (p > 0.05). In the order hand, the treatment with PAb gel increased the total amount of collagen, specially type I collagen, and significantly decreased type III collagen when compared to ZA group, corroborating our previous findings on osteoblast analysis.

On Raman spectrometry, it was seen that ZA reduced the amount of bone mineralization, seen by MTMR ratio. Maxillary bones, of animals from ZA group, showed higher CTPR, indicating increased solubility. These bones also presented increased mineral maturity ratio, compatible to older bones. Collagen crosslink ratio was significantly higher in ZA group, corroborating collagen analyses, previously described. BRONJ was reduced bone remodeling (HA carbonate/amide I ratio) suggested by the reduction on osteoblast cell count. PAb gel reversed all the findings caused by BRONJ on bone tissue (Table 2).

**Table 2:** Effect of ATV on Raman spectroscopy parameters for bone substrates.

Ratio	CONTROL	ZA	PAb gel
MTMR	2.576 ± 0.453	0.502 ± 0.049*	2.073 ± 0.266^#^
CTPR	0.825 ± 0.136	1.694 ± 0.206*	0.464 ± 0.090^#^
Mineral maturity	1.697 ± 0.345	3.522 ± 0.703*	1.215 ± 0.055^#^
Collagen crosslinks	1.420 ± 0.940	3.640 ± 1.040*	0.70 ± 0.400^#^
HA carbonate/amide I	1.098 ± 0.080	0.420 ± 0.050*	1.060 ± 0.065^#^

Values are presented as mean ± SEM.

*ZA* zoledronic acid, *PAb* polyssacharide from *Agaricus blazei*, *MTMR* mineral-to-matrix ratio, *CTPR* carbonate-to-phosphate ratio, *HA* hydroxyapatite.

*Indicates difference when compared to the Control group (P < 0.05).

^#^Indicates difference when compared to ZA group (P < 0.05). ANOVA and Tukey.

## Discussion

This study demonstrated that the BRONJ model in rats exhibited characteristics such as the exposure of necrotic bone without mucosal healing and, notably, a decrease in the number of viable osteocytes with an increase in empty lacunae^6,16,26,27^, mirroring the features of BRONJ lesions in humans^28^. The BRONJ model also had a detrimental impact on osteoblast count and function by inhibiting Wnt signaling, thus confirming reduced bone remodeling. Moreover, ZA (zoledronic acid) led to a reduction in total collagen, especially type I collagen in bone tissue, as supported by collagen degradation observed in Raman spectrometry. The bone tissue subjected to the BRONJ model also exhibited reduced mineral content and increased solubility, resembling the characteristics of aged bone. Importantly, the use of PAb (polysaccharide from *Agaricus blazei*) counteracted all the findings observed in the ZA group. To the best of our knowledge, this is the first instance of reporting the local effect of a polysaccharide from Agaricus blazei in a BRONJ model in rats.

It was seen that, PAb gel protected bone subjected to the BRONJ model, reducing bone exposure and increasing the count of viable osteocyte cells. Bone necrosis is a hallmark of MRONJ. While osteocyte death occurs as a natural part of the skeletal life cycle, the suppression of bone resorption by bisphosphonates (BPs) has been suggested to lead to the accumulation of dead osteocytes. Prolonged exposure to BPs may also reach cytotoxic levels for osteocytes. These accumulated necrotic osteocytes release high levels of Damage-associated molecular patterns (DAMPs), further promoting inflammation that results in damage to oral soft and hard tissues^29^. Considering the effects of beta-glucan on bone tissue, a model of steroid-induced avascular necrosis of the femoral head in rabbits has shown that this polysaccharide reduced empty lacunae and decreased osteocyte apoptosis. This was marked by a decrease in the expression of pro-apoptotic regulators Bax and Caspase-3, as well as an increase in the expression of the anti-apoptotic regulator Bcl-2. These findings suggest that Bax, Bcl-2, and Caspase-3 are involved in the anti-apoptotic effects of this polysaccharide^30^, which aligns with our own findings.

Here, we have demonstrated that PAb increased the number of osteoblast cells and enhanced their function by stimulating the Wnt pathway, as evidenced by increased levels of β-catenin and Runx2. Previous research has indicated that extracts from Agaricus blazei can promote the expression of genes associated with osteoblast activity and bone formation^31^. While the impact of bisphosphonates (BPs) on bone remodeling delay is linked to the development of BRONJ, their effect on osteoblasts is less explored^5^. High doses of BPs have been reported to arrest the osteoblast cell cycle and induce apoptosis, consequently reducing osteoblast lineage proliferation^5^. Our previous research has also demonstrated the detrimental effect of BRONJ on osteoblasts and the Wnt pathway^6,32,33^. However, the intriguing effect of PAb gel on osteoblasts observed in this study can be attributed to its ability to enhance osteoblast adhesion, growth, and proliferation^34^. Mushroom beta-glucan has also been found to stimulate Wnt/beta-catenin signaling in wound and healing models^35^. In bone tissue, beta-glucan has been shown to reduce osteoclast numbers and concentrations of inflammatory markers like IL-1β and TNF-α^4^, as well as downregulate RANKL and upregulate osteoprotegerin (OPG)^36,37^. The reduction in the proliferation and activity of osteoclasts induced by beta-glucans undoubtedly supports osteoblast function, as evidenced by the increase in BALP levels^13^, which aligns with our own results. Collectively, these findings demonstrate that PAb gel has the potential to restore bone remodeling, which is often reduced during the use of ZA.

In addition to the quantity of bone, the quality of this tissue is of paramount importance. PAb gel not only improved the quantity but also the quality of collagen. Collagen, as the primary component of the extracellular matrix^38^, serves as a structural framework in tissues during the healing process, influencing cell proliferation and migration^39^. Type III collagen plays a crucial role in the initial stages of the healing process, where it is synthesized by fibroblasts in the granulation tissue^40^. As the wound matures and closes, type III collagen is broken down, and the synthesis of type I collagen increases^40^. Soundia et al.^41^ demonstrated that necrotic bone exhibits disorganization in the collagen network with a predominance of type III collagen. The effects of beta-glucan on collagen have been previously demonstrated in wound and healing models, wherein it enhances collagen deposition and organization^42^. The improved quality of collagen after using PAb gel was further confirmed by assessing the collagen crosslink ratio through Raman spectrometry. Lower values of this ratio indicate a lesser degree of structural deterioration in collagen, making it a valuable tool for evaluating collagen quality and structural integrity in bone^23^.

In this study, the improvement in bone quality after PAb use was further verified through Raman spectrometry, as it led to an increase in mineral content and a reduction in fragility, resulting in a more resilient and robust bone tissue. The increase in mineral-to-matrix ratio (MTMR) indicates a higher mineral content in the bone tissue, directly enhancing bone strength and making it more resistant to fractures^42^. A higher carbonate-to-phosphate ratio (CTPR) signifies that phosphate positions in the apatitic lattice, which are susceptible to ionic substitution, often referred to as "B-type" carbonate substitution, are associated with reduced bone solubility. Lastly, mineral maturity reflects the progressive transformation of non-apatitic domains into well-crystallized apatite, and it can be influenced by alterations in bone remodeling, such as the use of bisphosphonates (BPs), which tends to increase mineral maturity^22^. While Raman spectroscopy is widely used in various fields, including the detection of tumors in biology and medicine, its application in discriminating BRONJ has been reported only infrequently. Our findings are consistent with some authors^43^ and contradicted by others^44^, highlighting the need for additional studies to more comprehensively evaluate BRONJ lesions using Raman spectrometry.

Despite the beneficial effect of PAb gel in treating BRONJ, it is important to note that this study has certain limitations. The role of Wnt signaling warrants further in-depth investigation, and in vitro assays must be conducted to determine how PAb interacts with osteoblasts. Given the multifactorial etiology of BRONJ, additional aspects such as the potential anti-inflammatory, antimicrobial, and angiogenic effects of PAb should be examined in the near future.

## Conclusion

In conclusion, the findings of this study suggest that the PAb gel derived from Agaricus blazei may have a mitigating effect on bone necrosis in the model of Bisphosphonate-Related Osteonecrosis of the Jaw (BRONJ). This study underscores the potential of PAb as a pharmacological tool to support or prevent BRONJ therapy. Nevertheless, further investigations, including clinical trials, are essential to validate its efficacy and safety in patients.

## Data Availability

The datasets generated and/or analysed during the current study are available in the Open Science Framework repository, at Identifier: 10.17605/OSF.IO/3MA5K.
